# Building adjustment capacity to cope with running water in cultured grass carp through flow stimulation conditions

**DOI:** 10.1038/s41598-024-59270-6

**Published:** 2024-04-14

**Authors:** Qingrong Xie, Li Wang, Shengfa Yang, Wei Yang, Jiang Hu, Wenjie Li, Xianbing Zhang, Ziwei Chen

**Affiliations:** National Inland Waterway Regulation Engineering Research Center, Chongqing Jiaotong University, Xufudadao 66, Chongqing, 400074 China

**Keywords:** Flow stimulation, Cultured grass carp, Adjustment capacity, Running water, Migration strategy, Sustainability, Behavioural ecology, Ecosystem ecology, Freshwater ecology

## Abstract

The adaptability of cultured fish to complex flow conditions is crucial for their survival after being released into the wild. Running water in natural environments poses significant challenges for the proliferation and release of cultured fish. This study aimed to investigate the effects of flow stimulation on the adjustment capacity of cultured fish to cope with running water. The target fish were cultured grass carp. An annular flume was used to conduct tests on training and control groups. The results demonstrated an enhancement in the adjustment capacity of cultured fish following appropriate flow stimulation training. (1) The trained fish exhibited a heightened preference for low-velocity areas. (2) The trained fish displayed the ability to select a route characterized by low energy consumption, predominantly following the periphery of the low-velocity area. This suggested that an appropriate flow velocity could improve the sensitivity of training fish to water flow information, and their adjustment capacity to cope with running water improved to a certain extent. A higher adjustment capacity allowed them to process flow rate information rapidly and identify a migration strategy with lower energy consumption. This study provides a useful reference for enhancing the survival rate of grass carp through stock enhancement initiatives and contributes to the sustainability of freshwater ecosystems.

## Introduction

**Figure 1:**
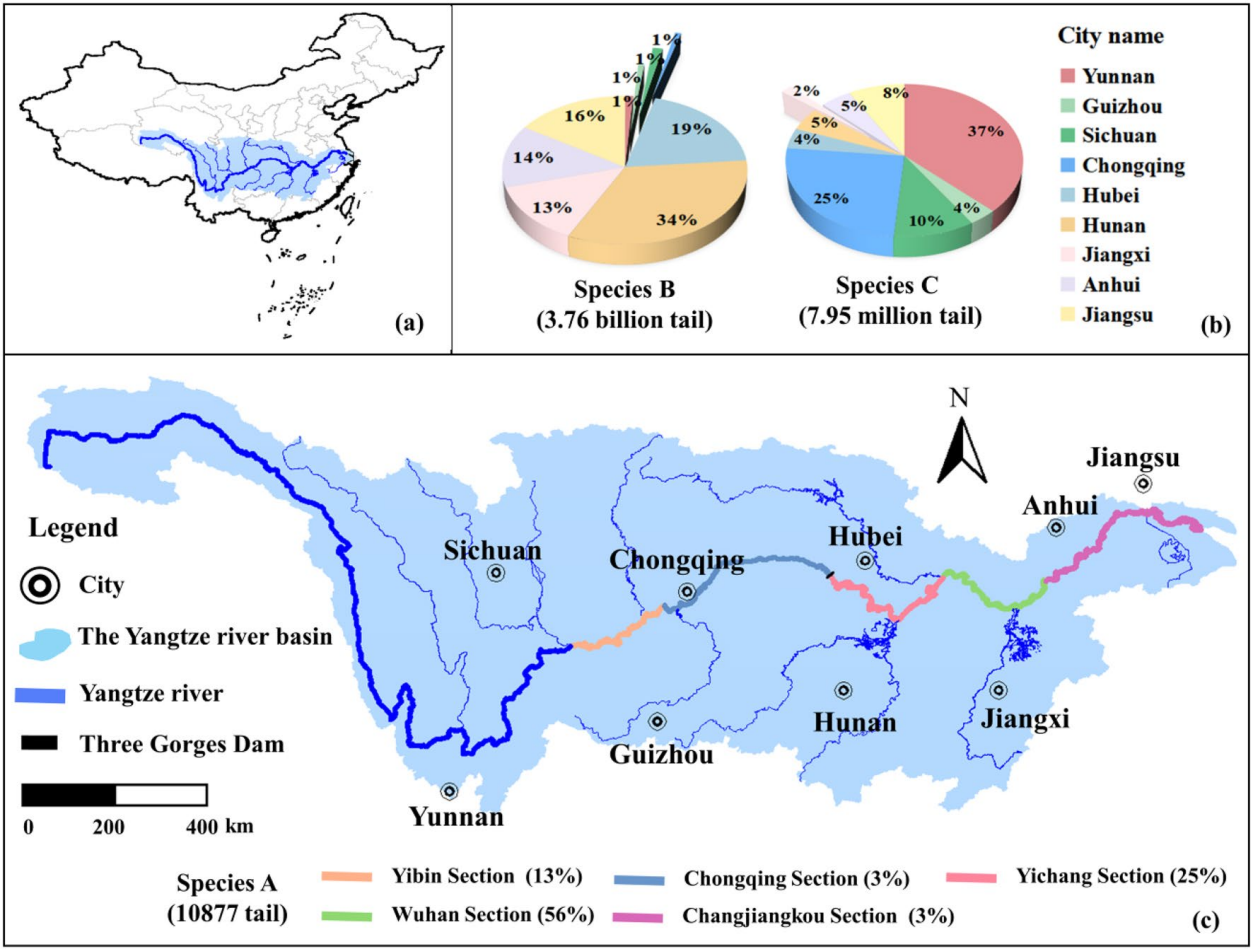
Location, distribution, and quantity statistics of stock enhancement initiatives of major cities in the Yangtze River Basin in 2019. The stock enhancement initiatives of three species are shown. (**a**) Location of the Yangtze River Basin in China. (**b**) Species B represents economic aquatic animals; species C represents rare and endemic aquatic animals. The two pie charts represent the proportion of fishtails released of species B and C in the proliferation and release plan of cities along the Yangtze River Basin, here, the different color legends represent cities. (**c**) Species A represents Chinese sturgeon and Yangtze sturgeon. Lines with different colors represent the different sections of the Yangtze River Basin. Prepared using ArcGIS 9.0 software (http://www.esri.com). This work is licensed under a Creative Commons by Attribution (CC BY-NC-ND) license.

Freshwater ecosystem is considered to be the most abundant and diverse system on the planet. However, it was experiencing a rapid decline in terms of biodiversity due to human activities, such as hydropower, shipping, flood control, agriculture, and urban development^1–5^. The Yangtze River, which is characterized by high species richness and endemism, is the longest river in China and the third longest river in the world. Unfortunately, over the past few decades, human activities and environmental changes have significantly accelerated the loss of fish biodiversity in the Yangtze River Basin. Various effective measures have been taken to protect freshwater biodiversity, including fishing bans, the establishment of natural resource conservation areas, and ecological scheduling^6–11^. Artificial breeding and stock enhancement are effective and expedient solutions for addressing declines in fish resources^12^, and have been successfully implemented in multiple countries such as France, Japan, the United States, the former Soviet Union, and Norway, among others. It has emerged as a promising strategy for future resource conservation and ecological restoration^13,14^. For instance, in recent years, several regions in China have conducted release activities to aid in the recovery of fish stocks. In 2019, the release of economically significant fish, particularly Four Major Chinese Carp species in the Yangtze River Basin, amounted to a population of 3.76 billion, as demonstrated in Fig. 1.

However, the effects of proliferation and release activities were not significant, as demonstrated by the low survival rate of the released species^15^. Previous studies have estimated that the contributions of proliferation and release to the wild population range between 0.45 and 1.9%, with a maximum of contributions less than 10%^16,17^. Reports from Japan, France, New Zealand, Australia, and other countries have shown that proliferation and release programs do not yield desirable results^18–20^. The low contribution rate could be attributed to several factors, including behavioral and health defects in artificially cultured species^21,22^, higher predation risks^23^, stocking density, and body size^24,25^. Notably, the complex flow conditions in natural environments, in contrast to still water in farms, may also contribute to for their low survival rates^26,27^. Complex water flow environments pose significant challenges to cultured fish, thereby contributing to their low survival rates^28^. Consequently, enhancing the adaptability of the released species to the water flow environment is crucial for promoting aquatic biodiversity.

Studies have demonstrated that the implementation of water flow stimulation to prompt the movement of fish in aquaculture farms led to enhancements in their swimming abilities, predation, avoidance, rheotaxis, and oxygen-carrying capacity^29–33^. It has been observed that the growth rate and food conversion efficiency of diverse fish species can be significantly enhanced by varying the intensities of water flow stimulation. Moreover, this stimulation leads to increased aerobic potential in both red and white muscles, as well as improved cardiac performance^34^ and ultimately improves swimming performance^35,36^. Moreover, appropriate exercise training through water flow stimulation has been utilized to enhance the swimming ability, growth, and immunity of fish^36–38^, and has been widely used in fish breeding and stock enhancement initiatives^39^. Although numerous studies have provided insights into the physiological effects of water flow stimulation on fish and their swimming abilities, there remains a dearth of research regarding the impact of water flow stimulation on the adjustment capacity to complex flow conditions in natural environments^40^. Therefore, it is imperative to conduct further investigations into the influence of flow velocity stimulation on the adjustment capacity of fish to cope with running water, thus aiding their proliferation and release.

It can be hypothesized that if exercise training builds adjustment capacity to cope with running water in cultured grass carp, it is of great significance to improve the field survival rate of grass carp in the stock enhancement initiatives, and can provide reference for the stock enhancement initiatives of other species. To test this hypothesis, a comparative test was designed between training group and control group, compared the differences in the ability to process of velocity information and energy consumption strategies between the two groups of test fish. Adjustment capacity text in training and control groups were performed in a recirculation flume. The entry frequencies, residence time in different flow velocity zones, and movement patterns of the two groups were studied. Finally, the effect of water flow stimulation on the adjustment capacity of cultured grass carp to cope with running water was discussed. The findings of the present study can inform river management and aquatic life protection.

## Methods

### Experimental fish and rearing condition

Grass carp (*Ctenopharyngodon idellus*), one of the Four Major Chinese Carps, was the main subject of this study. Since most of the grass carp fry released for into the wild are approximately 10–15 cm in length^15,41^, 50 tails healthy and alive grass carp juveniles with a body length of 10–13 cm, purchased from a fish farm in the Jiangjin District of Chongqing, were selected as experimental fish. Before starting the experiments, the purchased fish were reared in two 3 × 2 m cages (25 tails per cage) in Lizi Lake, Shuangfu Campus, Chongqing Jiaotong University for one week. During this period, the water temperature was 10 ± 1 °C, pH was 7.6–8, dissolved oxygen value was 6–7 mg/l, and lighting was natural light. Oxygen was continuously supplied pump to ensure the stable dissolved oxygen level, and dissolved oxygen was measured at 1-h intervals. Fish were fed with commercial floating pellet feed (crude protein ≥ 40%, fat ≥ 5%, crude fiber ≤ 6%, crude ash ≤ 16%) at 07:00 every day. After temporary rearing to adapt to the laboratory water quality environment, grass carps were subjected to flow stimulation training and adjustment capacity tests.

### Flow stimulation training experiment

**Figure 2:**
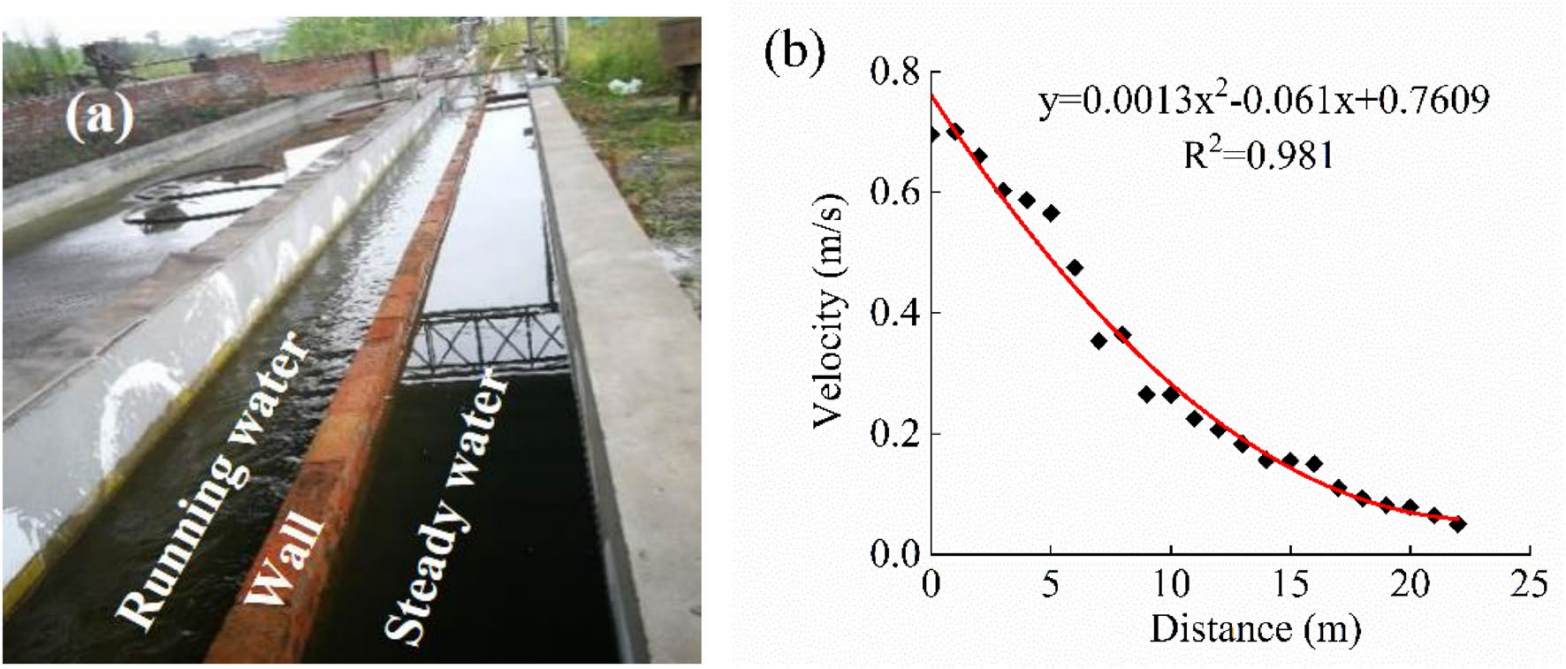
(**a**) Schematic diagram of the flow rate stimulation training flume and the control group static flume, the left side represents the running water, and the right side represents the steady water; (**b**) curve of the flow velocity at the central axis of the flume changing with distance, the initial position was the barrier net of the training flume.

Following a seven-day period of temporary rearing in cages, a three-week adjustment capacity training regimen was conducted on a group of thirty uniform-length grass carp. The experiment of flow stimulation training was carried out in two flumes, named the training flumes and the control flumes, each with dimensions of 22 m × 0.6 m × 0.5 m (length × width × height). McFarlane et al.^42^ reported that there was no significant difference in fish behavior between individual and group training; hence, 15 fish were trained together in running water to reduce the experimental duration. In contrast, another batch of 15 fish was placed in the flume under steady water conditions, as shown in Fig. 2a. The flow velocity of the training flume was regulated using two pumps at a total flow rate of 400 m^3^/h. Within the training flumes, the velocity of water in proximity to the pump was approximately 0.7 m/s, which was approximately 0.6 times the critical swimming speed of the experimental fish. The water velocity away from the pump was maintained at approximately 0.05 m/s, and the water depth was set at 55 cm. Figure 2b illustrates the distribution of flow velocity along the axis of the flume in the training group, which was measured using Nortek Acoustic Doppler Velocimetry, as previously described by Yan et al.^43^.

During the training period, feeding was conducted from 07:00 to 09:00 in the morning, with 19:00 to 21:00 designated as the rest period (flow velocity was 0 m/s) and the remaining 20 h designated as the training time. Both in training and contral group, the flume water temperature was maintained at 9 ± 1 °C, pH was 7.6–8, dissolved oxygen value was 6–7 mg/l, with natural light. Continuous oxygen supply by oxygen pump to ensure the stable dissolved oxygen level, and dissolved oxygen was measured at 1-h intervals. The morphological parameters of all 30 fish were measured before and after the experiment.

### Adjustment capacity test

**Figure 3:**
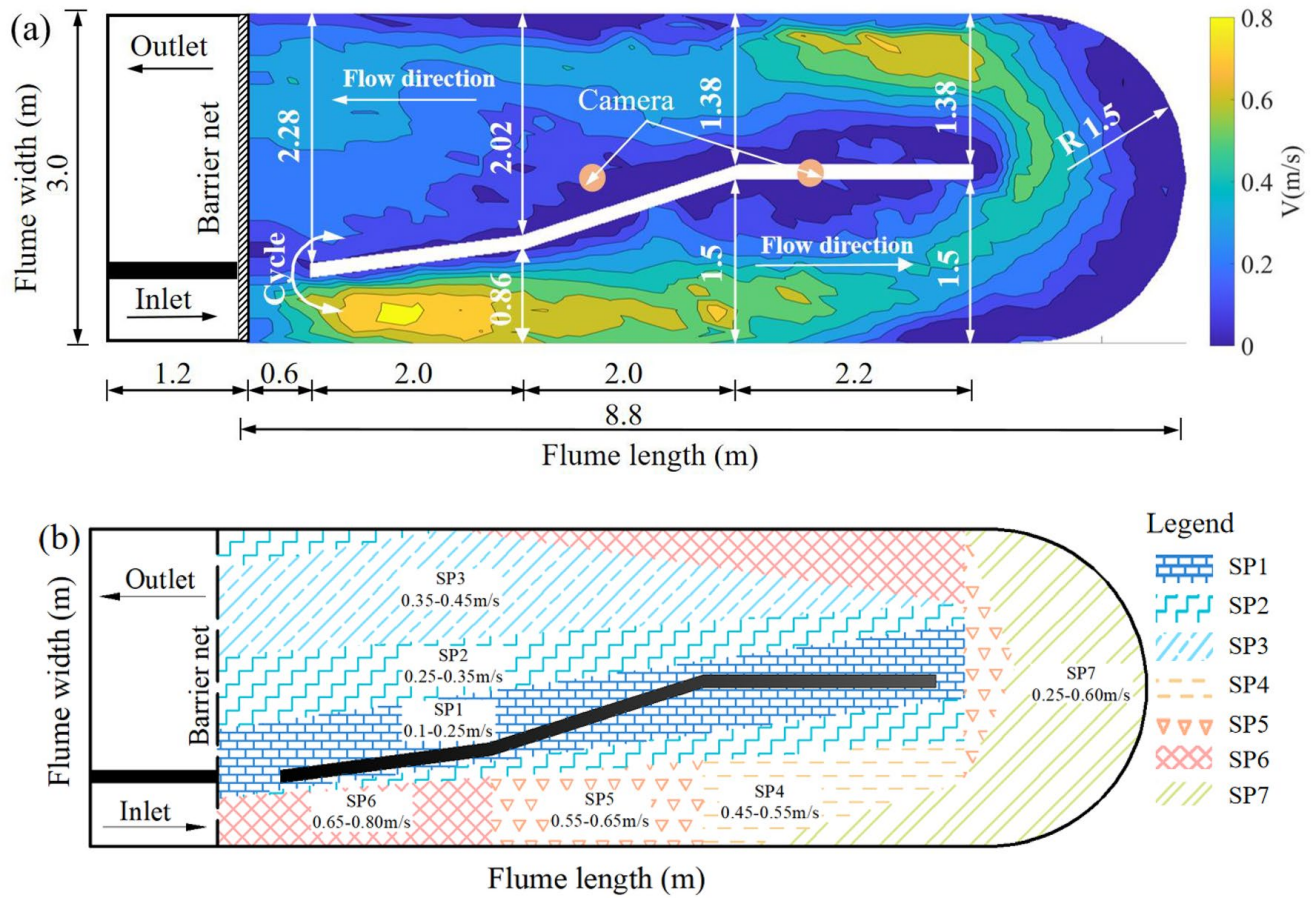
(**a**) Schematic diagram of circulating water channel for fish adjustment capacity training using water velocity stimulation. The bottom cloud map represents the velocity distribution in the circulating flume measured by Particle Image Velocimetry techniques. The Roman numerals in the figure represent the labeling of the dimensions (meter). (**b**) Flow velocity partition diagram. Different colors and different pattern fills represent different flow zones, refer to the legend in figure.

An adjustment capacity test was conducted in a recirculation flume with 10 m × 3 m × 0.6 m (length × width × height). An irregular retaining wall was set in the middle of the flume to change the flow area and generate velocity gradients (Fig. 3a). The velocity distribution in the recirculation flume was constant and measured using Particle Image Velocimetry techniques. Based on the velocity distribution in the flume, seven distinct velocity regions were identified to evaluate the preferences of grass carp for different velocities before adjustment capacity training (Fig. 3b). Specifically, the SP1 area, representing the flow velocity near the retaining wall and measured to be approximately 0.1–0.25 m/s, typically exhibited low flow velocities. In contrast, the SP2, SP3, SP4, SP5, and SP6 areas, corresponding to flow velocities ranges of 0.25–0.35 m/s, 0.35–0.45 m/s, 0.45–0.55 m/s, 0.55–0.65 m/s, and 0.65–0.8 m/s, respectively, were characterized by progressively increasing flow velocities. Additionally, the annular area of the flume exhibited a wide range of velocity distributions and was considered a transition zone that fish were not actively entering by fish. Consequently, this area was designated as the SP7 area and was excluded from the flowing analysis. During the experiment, the flume water temperature was maintained at 10 ± 1 °C, pH was 7.6–8, dissolved oxygen value was 6–7 mg/l, with natural light. Continuous oxygen supply by oxygen pump to ensure the stable dissolved oxygen level, and dissolved oxygen was measured at 1-h intervals.

**Figure 4:**
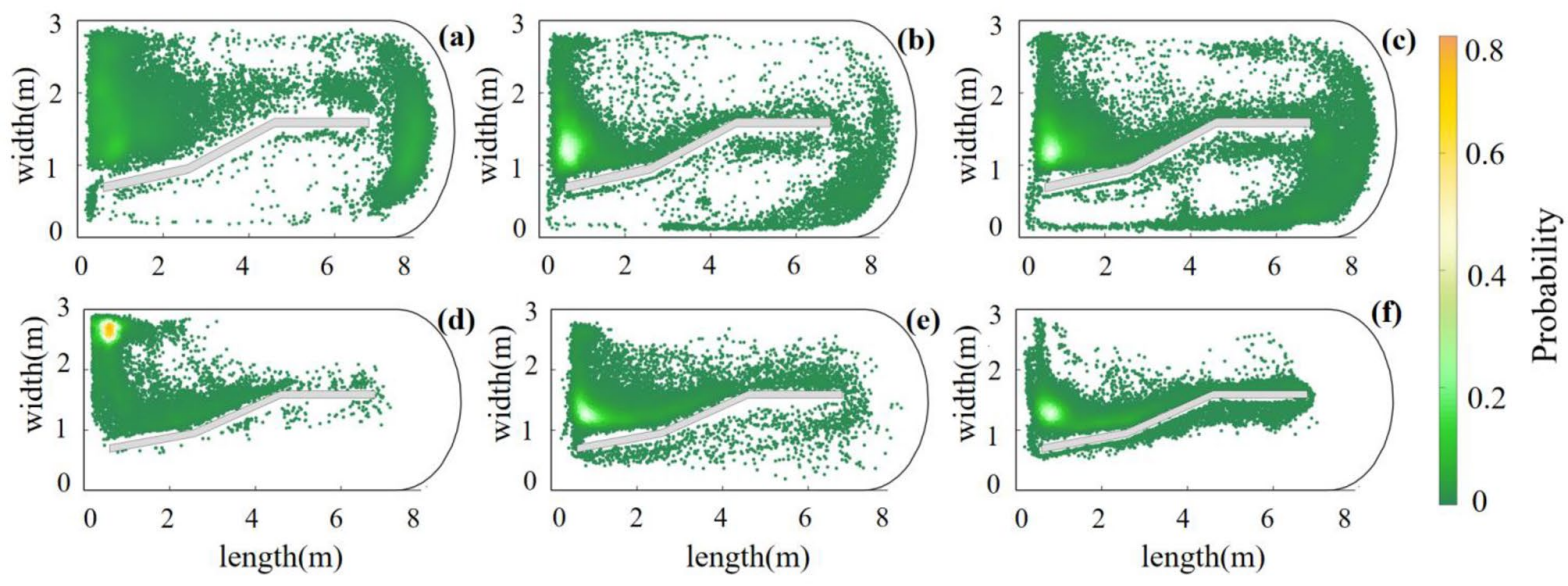
Track point nephograms of grass carp in each flow velocity area in the flume of the Cg and the Tg in periods T1, T2, and T3. The color scheme indicates the frequency of fish entering each flow velocity area, with orange-yellow representing higher frequency and dark green indicating lower frequency. Specifically, panels (**a**–**c**) respectively represent the 8th, 16th, and 24th h track distribution cloud maps of the Cg; (**d**–**f**) respectively represent those of the Tg.

High-definition cameras were positioned directly above the experimental flume at distances of 3.4 m and 5.4 m from the barrier net to capture the movement process of the experimental grass carp (the planar position was shown by the orange dots in Fig. 4). The camera utilized a resolution of 1200 × 720 pixels and a frame rate of 25 frames/s, while the lens was directed towards the water surface. An LED light source was installed above the flume to ensure a uniform light background and to reduce shadows. The training group (T-group, abbreviated as Tg) consisted of 15 grass carps stimulated by water flow, whereas the control group (C-group, abbreviated as Cg) consisted of 15 grass carps in steady water tanks. The fish were fasted for 24 h prior to the experiment. During the experiment, the fish (n = 15 in each group) were placed in the circulation tank in the low-flow rate area to adapt for 10 min, after which two cameras were activated to record the trajectory of the fish for 24 h. Individual swimming trajectories of 15 fish in each group were obtained from the video data to analyze the results.

### Data analysis

Upon completion of the experiment, the motion trajectory of the grass carp was identified and extracted using image processing technology. To circumvent the issue of data redundancy resulting from extensive video data, we analyzed three typical time nodes that were deemed representative of the overall dataset. These time nodes included the 8th hour of the initial stage (T1), the 16th hour of the intermediate stage (T2), and the 24th hour of the end stage (T3) of the experiment, with 15 fish per group. Experiments have demonstrated that the entry frequency and residence time of fish in different flow velocity regions can be used to evaluate the swimming ability of fish^44^. Therefore, in this experiment, the entry frequency of the test fish in the different flow velocity regions and the residence time in different flow velocity regions were selected as evaluation indices of flow tendency. Correction and calibration of video images were conducted, followed by the calculation of two parameters, including the entry frequency of the fish in each flow area (*P*_*Fn*_) and the percentage of residence time in each flow area (*Pt*).

Based on the divided flow velocity regions mentioned above, the formula for the calculation of the frequency percentage of grass carp entering each flow velocity region is defined as:

$${P}_{{F}_{n}} = \frac{{F}_{n}}{F}\times 100\%$$

$$F=\sum_{1}^{n}{F}_{n}\left({\text{n}}=\mathrm{1,2},\cdot \cdot \cdot \cdot \cdot \cdot ,7\right)$$

where ${P}_{{F}_{n}}$ is the percentage frequency (%) of the experimental fish entering each velocity zone, *Fn* is the frequency of the experimental fish entering each velocity area, and $F$ is the sum of the frequencies of the experimental fish entering each velocity area.

The formula for calculating the percentage of residence time of grass carp at different flow rates is defined as:

$${P}_{t} = \frac{t}{{t}_{0}}\times 100\%$$

where ${P}_{t}$ is the percentage of residence time of the experimental fish in different flow velocity areas (%), ${t}_{0}$ is the total residing time(s) of the experimental fish in each velocity area, $t$ is the total video time of choice for each fish (fixed at 3600 s).

The swimming energy consumption of fish varies because of different water environments along swimming paths. Previous studies have found that the swimming energy consumption of fish is closely related to the flow rate of the water, fish body length, body weight, and other factors^26,27,45^. In 2005, Eva C developed a new model to predict total swimming cost, which was highly consistent with the experimental demands of this study^26^. Therefore, this model was used to predict the energy consumption of the three swimming paths of grass carp. The model is defined as follows:

$${log}_{10}COT=0.96{log}_{10}M+0.23{log}_{10}\overline{u }+0.67{log}_{10}T-1.85$$

where $COT$ is the swimming cost (mg O_2_·h^-1^), $M$ is the wet weight of fish (g), $\overline{u }$ is the average flow rate (cm/s); $T$ is the temperature, calculated as 10 °C according to the test conditions.

Conventional statistical analysis of experimental data was performed using Excel software and Origin 2023 software for Two-Way ANOVA analysis of variance to test for significant differences between the Tg and Cg. The level of statistical significance for all analyses was set at *p* < 0.05. Tukey tests were used to assess the progression of each group. Extraction and analysis of the motion trajectories of grass carp were recorded in the experiment using Programming Language, supplemented by free image analysis and modeling software Tracker based on the Open Source Physics (OSP) Java framework.

### Ethics approval and consent to participate

After the experiment, healthy experimental fish were cultured in the laboratory, and unhealthy fish were immersed in solutions of MS 222 for euthanasia treatment. The Water Conservancy and Water Transportation Laboratory in Chongqing Jiaotong University approved this experiment study (SYXK (Chongqing) 2021–0005). We confirm that all fish in the experimental protocols were reared and handled following the ethical guidelines and legal requirements under permission from the Laboratory Animal-Guideline in China (GB/T 39760-2021) and the AVMA Guidelines for the Euthanasia of Animals (2020). All experiments were performed following ARRIVE guidelines and regulations.

## Results

### Trajectory distribution at different times

The track distribution of the experimental fish in the Cg and Tg during the 8th, 16th, and 24th hourly periods representing the initial, intermediate, and final phases of the experiment, respectively, was statistically analyzed. The trajectories of the Cg were distributed across various flow velocity areas in the flume, and some individuals in this group were observed to recklessly enter areas with high flow velocities, resulting in them being washed back to their starting position by the strong current (Fig. 4a–c). However, grass carp in the Tg exhibited a distinct preference for motion tracks, primarily remaining in areas characterized by low flow velocities, such as SP1 and SP2 (Fig. 4d–f). They hardly accessed the areas with high flow velocity and were only occasionally brought to the edges of the areas with high flow velocity by water flow.

### Entry frequency percentage of different flow velocity areas

**Figure 5:**
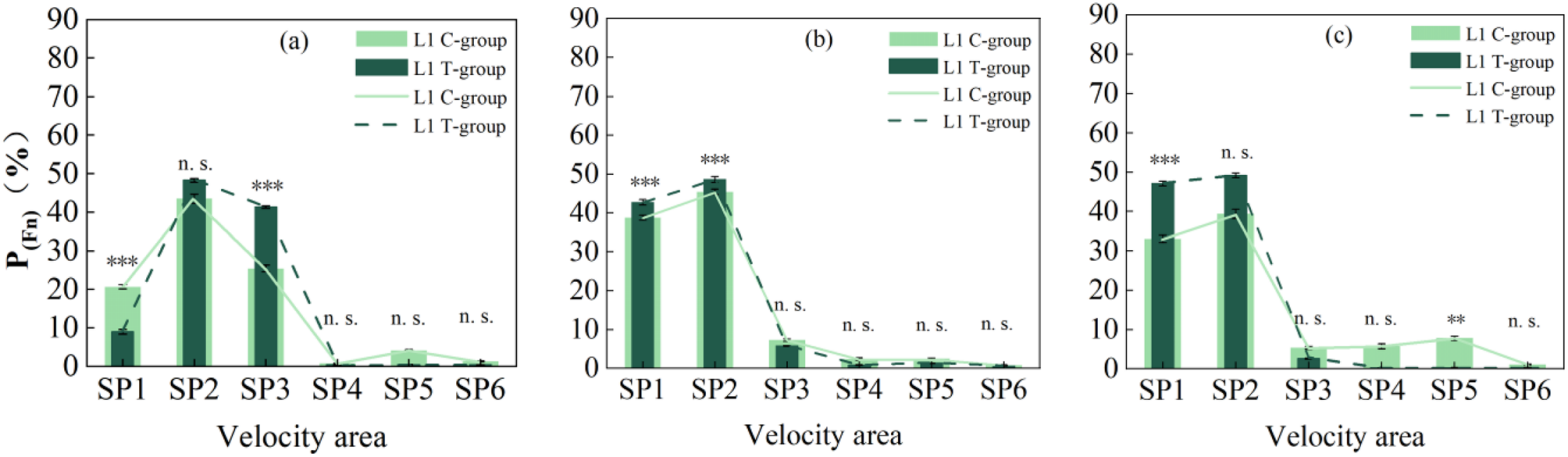
Percentage entry frequency of grass carp entering each flow velocity area in the Tg and the Cg at different times. The dark dotted line indicated the experimental group, and the light solid line indicated the control group. The difference test, in the Tg and the Cg at the three time periods are statistically significant (*p* < 0.05). (**a**) T1, the 8th hour of the experiment; (**b**) T2, the 16th hour of the experiment; (**c**) T3, the 24th hour of the experiment. (*p < 0.05, **p < 0.001, ***p < 0.0001, n. s. *p *> 0.05).

The entry frequency of grass carp in each velocity area by the two groups was analyzed according to the selected experimental video images in three typical periods, as shown in Fig. 5a–c. The study reveals that the SP2 velocity area exhibits the highest entry frequency during both the Tg and Cg, indicating a preference for this velocity area by both groups of fish, which remains stable over time. For the SP1 area with the lowest velocity, at time T1, the entry frequency of Cg was significantly higher than that of the Tg (*p* < 0.0001). By time T2, the entry frequency of both groups of grass carp into the SP1 area significantly increased compared to T1, with the Tg shown a significantly higher frequency than the Cg (*p* < 0.0001). The status of both groups of grass carp at time T3 shown little change compared to T2; the proportion of the Tg in the low-velocity area SP1 slightly increased, significantly higher than that of the Cg (*p *< 0.0001), while the proportion of the Cg in SP5 increased, significantly higher than that of the Tg (*p *< 0.001). Regarding the SP3 area with moderately flow velocity, at time T1, the entry frequency of the Tg was significantly higher than that of the Cg (*p *< 0.0001). By time T2, the entry frequency of both groups of grass carp into the SP3 area significantly decreased compared to T1, with the Cg surpassing the Tg, but the difference was not significant (*p *< 0.0001). The status of both groups of grass carp at time T3 shown little change compared to T2; the proportions of both groups in SP3 slightly decrease, with no significant difference between the two groups (*p *< 0.0001). The entry frequencies of both groups of grass carp in the higher-flow SP4, SP5, and SP6 areas remain consistently low (Table 1), with the Tg shown lower entry frequencies than the Cg, but the difference was not significant (*p *> 0.05). Throughout the experiment, both the Tg and Cg transition from a preference for the SP2 and SP3 areas (T2, T3) to a preference for lower-velocity SP1 and SP2 areas, with the Tg demonstrated a marked preference for low-velocity areas and avoidance of high-velocity areas (SP4, SP5, SP6).

**Table 1:** Percentage of entry frequency of grass carp in Cg and Tg at T1, T2, and T3 in SP4, SP5, and SP6 high flow areas.

Fish length	Velocity area	Cg-P_Fn(T1)_ (%)	Tg-P_Fn(T1)_ (%)	Cg-P_Fn(T2)_ (%)	Tg-P_Fn(T2)_ (%)	Cg-P_Fn(T3)_ (%)	Tg-P_Fn(T3)_ (%)
10~13 cm	SP4	0.73	0.35	2.38	0.78	5.70	0.26
	SP5	4.07	0.39	2.43	1.29	7.78	0.29
	SP6	1.26	0.35	0.79	0.57	1.10	0.34

Tg represented the training group, Cg represents the control group.

### Percentage of residence time in each velocity area

**Figure 6:**
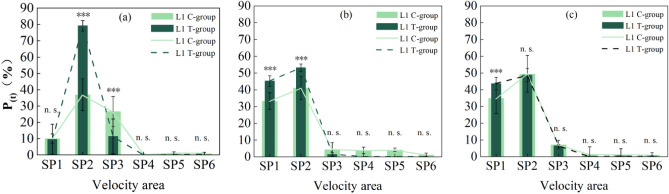
Percentage of residence time of grass carp in the Tg and the Cg at different time points in each velocity area. The dashed line indicates the Tg and the solid line indicates the Cg. The difference test, in the Tg and the Cg at the three time points are statistically significant (*p *< 0.05). (**a**) T1, the 8th hour of the experiment; (**b**) T2, the 16th hour of the experiment; (**c**) T3, the 24th hour of the experiment. (*p < 0.05, **p < 0.001, ***p < 0.0001, n. s. *p *> 0.05).

Similarly, experimental video images of three typical periods were selected to analyze the percentage of residence time of grass carp in each flow velocity area. The results were shown in Fig. 6a–c. The study revealed that both the Tg and Cg exhibited the longest residence time in the SP2 flow velocity area at all three time points, indicated a preference for this velocity area by both groups of fish. For the area with the lowest velocity, SP1, at time T1, there was little difference in residence time between the Tg and Cg (*p *> 0.05). However, by time T2, the residence time of both groups of grass carp in the SP1 area significantly increased compared to T1, with the Tg shown a significantly higher residence time than the Cg (*p *< 0.0001). The status of both groups of grass carp at time T3 shown little change compared to T2, with the Tg still significantly higher than the Cg (*p *< 0.0001). Regarding the SP3 area with moderately flow velocity, at time T1, the residence time of the Cg was significantly higher than that of the Tg (*p *< 0.0001). However, by time T2, the residence time of both groups of grass carp in the SP3 area significantly decreased compared to T1, with no significant difference between the two groups (*p *> 0.05). The status of both groups of grass carp at time T3 shown little change compared to T2, with both groups shown a slight increase in proportion in SP3, and no significant difference between the two groups (*p *> 0.05). The residence time of both groups of grass carp in the higher-velocity SP4, SP5, and SP6 areas consistently remained the lowest (Table 2), with the Tg exhibiting lower residence time than Cg, although the difference was not significant (*p *> 0.05). Throughout the experiment, both the Tg and Cg transitioned from a preference for the SP2 and SP3 areas (T2, T3) to a preference for the low-velocity SP1 and SP2 areas, with the Tg demonstrated a marked preference for low-velocity areas and avoidance of high-velocity areas (SP4, SP5, SP6).

**Table 2:** Percentage of residence time of grass carp in SP4, SP5, and SP6 at periods T1, T2, and T3 in Cg and Tg.

Fish length	Velocity area	Cg-Pt_(T1)_ (%)	Tg-Pt_(T1)_ (%)	Cg-Pt_(T1)_ (%)	Tg-Pt_(T2)_ (%)	Cg-Pt_(T3)_ (%)	Tg-Pt_(T3)_ (%)
10~13 cm	SP4	0.29	0.01	1.04	0.29	3.84	0.01
	SP5	1.03	0.02	1.16	0.39	4.23	0.01
	SP6	0.83	0.00	1.06	0.29	1.16	0.08

Tg represented the training group, Cg represents the control group.

### Moving patterns of upstream migration

#### Passage route

**Figure 7:**
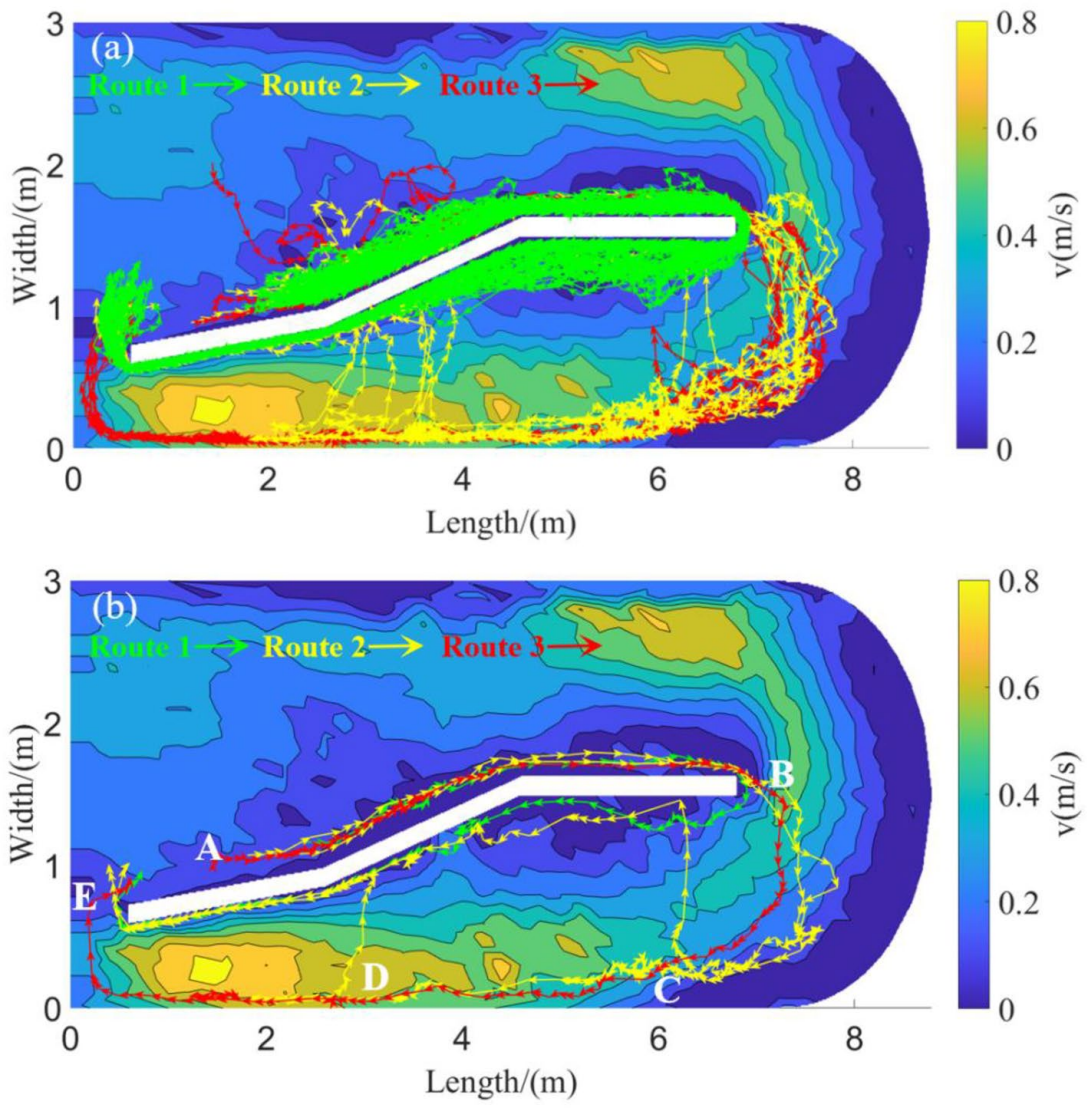
The experimental fish successfully completed the migration path of cycle selection. (**a**) All typical routes; (**b**) Three typical routes extracted.

The tracking of the motion trajectory indicated that the motion pattern distribution of all experimental grass carp in the two groups completed the circular motion (Fig. 7a). This study defines a counter current upward cycle as starting from point A and returning to point E through a combined path of B, C, and D. Three distinct routes were extracted from the motion trajectory and are represented by the red, green, and yellow arrows in Fig. 7b. Route 1 involved swimming beginning at point A, close to the middle retaining wall of the flume, turning at the end of the retaining wall (point B), while still close to the other side of the retaining wall, and finally returning to the starting point through the circulation port (point E). Route 2 involved swimming close to the retaining wall in the middle of the flume and being carried by the water flow to the peripheral side wall of the annular area of the flume, after which the fish moved towards the circulation port while avoiding high-flow-rate areas until reaching the first-choice point C. The fish then followed the low-flow-rate area near the retaining wall to complete the cycle. Route 2 could also manifest as the fish continued upstream along the peripheral side wall of the flume after passing the first-choice point C, then struggled to return to the middle of the flume at the second-choice point D near the retaining wall, and finally completed the cycle by following the retaining wall through circulation point E. Route 3 was similar to Route 2 but involved swimming close to the outer wall of the flume through the first-choice point C, then going through the second-choice point D by effort before finally completing the cycle by following the outer wall of the flume to point E of the circulation port.

**Figure 8:**
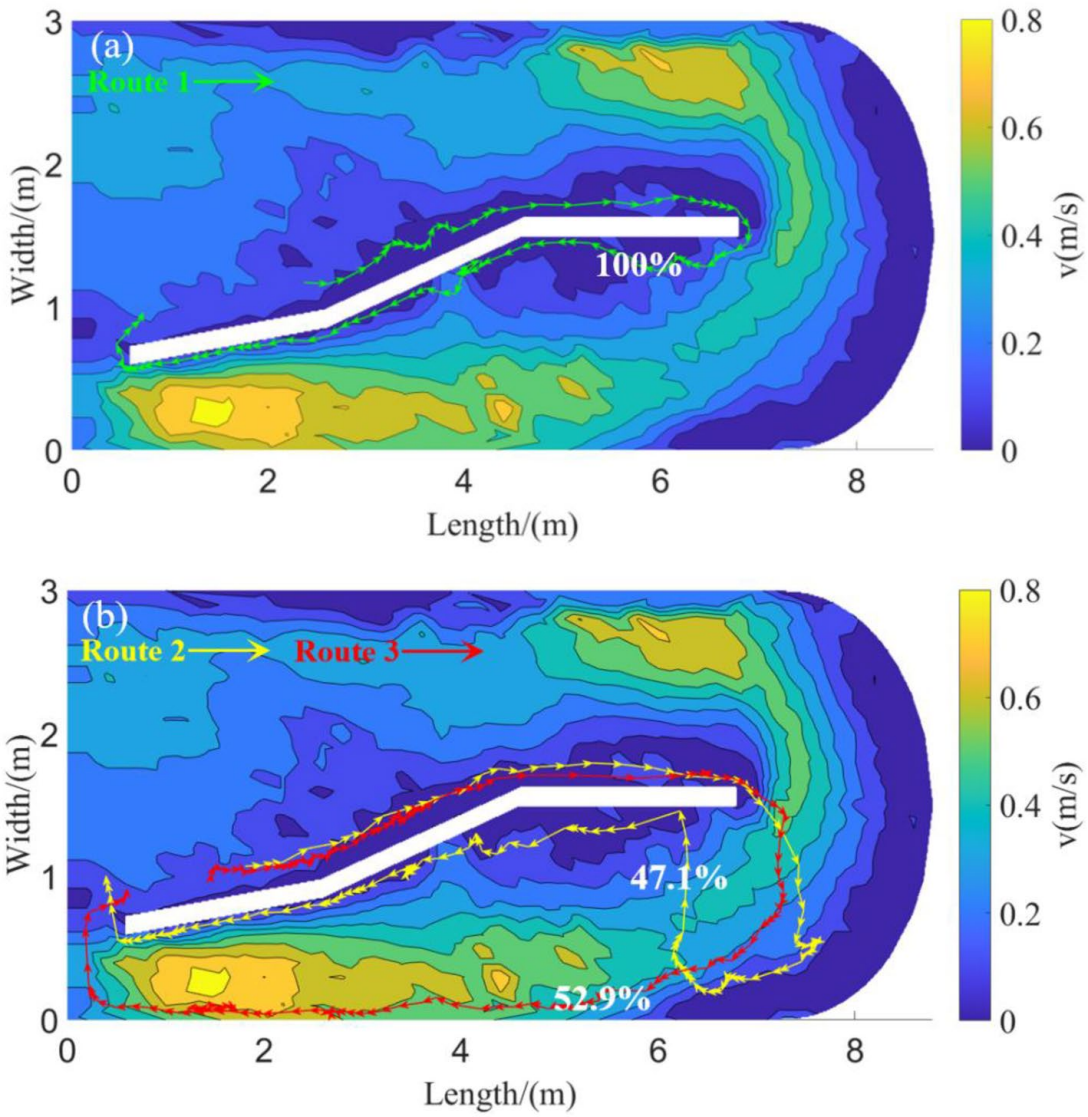
Schematic diagram and proportion of exercise mode selection of grass carp in Tg and Cg. (**a**) Moving pattern diagram of the Tg; (**b**) moving pattern diagram of the Cg.

According to the statistics of the percentage of route selection swimming of grass carp in the two groups (Table 3 and Fig. 8), the proportions of fish choosing routes 1, 2, and 3 in the Tg were 100, 0, and 0%, respectively, while the corresponding proportions of fish in the Cg were 0, 47.1, and 52.9%. Therefore, the main path selection for grass carp by the Tg was route 1, whereas routes 2 and 3 were selected by the Cg.

#### Cost of transport

**Figure 9:**
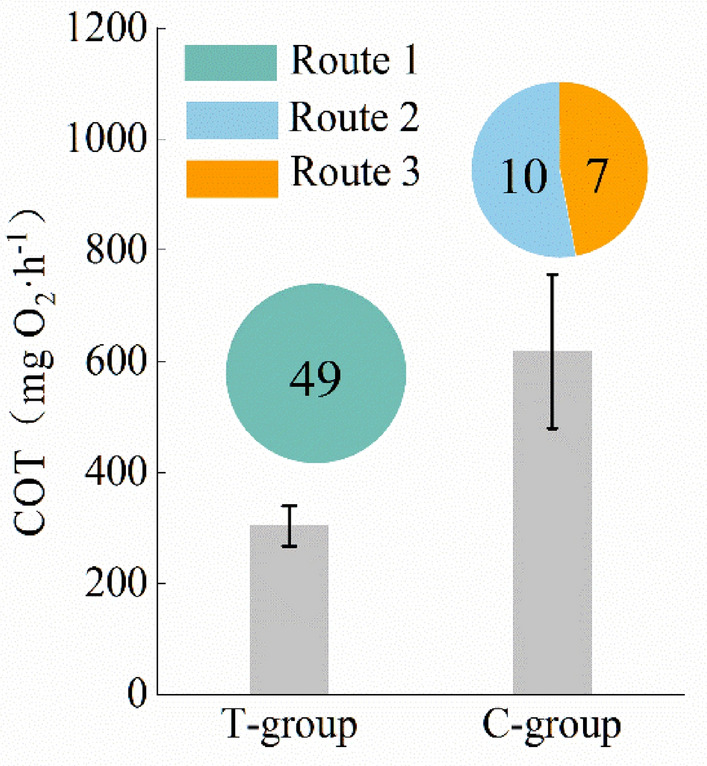
Exercise energy consumption statistics of three moving patterns of grass carp in Tg and Cg. The bar chart represents the motion energy consumption of each route (mean ± SE). The pie chart represents the number of times the corresponding motion mode was selected for each operating condition.

The results of the exercise energy consumption of grass carp in the two groups during upstream migration are shown in Fig. 9. The results demonstrated that grass carp in the Tg completed a significantly higher number of cycles (49) than those in the Cg (17) and exhibited a preference for the migration route with the lowest exercise energy consumption. Conversely, the Cg displayed higher exercise energy consumption, selecting routes 2 and 3 to complete the cycle. Compared with the average energy consumption of the passage route in both groups, the energy consumption of the migration mode of the trained fish was considerably lower (approximately 300 mg O_2_·h^−1^), representing only half of the energy consumption observed in the Cg. These findings suggest that training can enhance the exercise efficiency of grass carp during upstream migration as evidenced by their ability to choose a more energetically efficient route.

**Table 3:** The proportion of grass carp in the three swimming modes of the Tg and the Cg.

Group	Fish body length Means (cm)	Total number of route (time)	Moving patterns		
			Route 1 ratio (%)	Route 2 ratio (%)	Route 3 ratio (%)
Tg	10~13	49	100	–	–
Cg	10~13	17	–	47.1	52.9

Tg represented the training group, Cg represents the control group.

Tip:'–' indicates that the group of grass carps were not choose the corresponding moving patterns.

## Discussion

### Processing of velocity information

This study investigated the influence of water flow stimulation on the adjustment capacity of cultured fish to cope with running water. During the initial stage of the trial, both the Tg and Cg grass carp demonstrated a greater inclination to enter the SP2 and SP3 areas (Figs. 4 and 6), and towards the end of the experiment, the two groups exhibited a preference for the SP2 and SP1 areas. During the entire experiment, the entry frequency and residence times of the two groups were lower in the high flow velocity area (Tables 1 and 2). Notably, the change in entry frequency of the Tg was more significant compared to that of the Cg.

The results indicated that the adjustment capacity training method using flow velocity stimulation could enhance the ability of grass carp to process water flow information and enable them to distinguish and avoid high-flow velocity areas. Test fish exhibited a preference for low flow velocity areas, particularly the SP2 (0.25–0.35 m/s) area, which was consistent with previous conclusions^46,47^. The degree of preference was greater in the Tg than in the Cg, which could be attributed to prior training during the flow rate stimulation tests. Previous studies have consistently supported the notion that fish utilize fine-scale water velocity and turbulence as navigation cues for their movement behavior^48,49^, which aligns with the findings of this study. Fish can integrate water flow information in their natural environment, enabling them to execute a series of coordinated movements from one location to another, a process described by Burt as a self-centered cue employed by fish^50^. The improved flow tendency of trained fish can facilitate the rapid identification of low-velocity areas conducive to habitat selection^51,52^, foraging, avoiding predators^53^, and survival in the wild. It is anticipated that the survival rates of proliferation and release can be significantly improved in the future by the implementation of this strategy.

### Energy consumption strategy

Previous studies have shown that high turbulence increases the cost of fish movement owing to an increase in water flow resistance, leading to higher energy consumption^49,54^. Fish can optimize their energy costs and avoid physical fatigue by sensing and processing water flow information and making choices regarding their movement paths. Our experimental findings indicated that grass carp tended to choose a movement path that involved less energy consumption near the sidewall of the retaining wall after being trained by flow stimulation (Fig. 9 and Table 3). This finding is consistent with previous studies suggesting that fish tend to swim closer to sidewalls^55–57^. Additionally, it has been reported that migrating salmon breeding in lakes tend to spread along coastlines and eventually move to deeper waters^58^. Young anadromous fish that migrate from the middle of a river to the bank and then to the sea tend to follow similar patterns^51^. Juvenile silver carp tend to choose a sidewall with a low velocity to enter tributaries upstream of the separation zone of a channel confluence^52^. In addition to inland species, pelagic species exhibit a preference for coastal waters during migration. The Tagging of Pacific Pelagics Program (TOPP) was applied to five shark species, and its tracking results confirmed this conclusion^59^.

The 'Wall-following' behavior seems to explain this phenomenon. For a long time, there are two main hypotheses for the explanation of fish's wall-following behavior, namely, finding resources and/or constructing spatial reference for safety or explore^60,61^. They acquire spatial information by perceiving lateral line system, visual cues or physical contact, which is helpful for foraging, avoiding enemies and finding directions^60–62^. In this study, the spatial environment was the same and there was no predator, so the explanation of ‘wall-following’ behavior can only be the ability to explore spatial information. Due to the existence of different flow velocity areas in the flume, the stress effect on fish is different. The ‘wall-following’ behavior enables them to actively avoid the area with high pressure (high flow velocity area in this experiment)^60^, and select the low flow velocity area near the retaining wall (Fig. 4), in which the training group performed more obviously. This is also a model describing the energy saving of fish, because high flow rates are often accompanied by higher swimming costs^26,63,64^. In future studies, it is necessary to carry out in-depth research to discuss the effect of exercise training on fish wall-following behavior.

Finally, there is a need to address the potential for further improvements in the current experiment. While our research focused solely on grass carp, one of the major Chinese carp and the most widely cultured fish in the world, the sample size was relatively limited. Future studies should consider expanding this research to include other species. Furthermore, the energy consumption for the fish movement was calculated without using advanced fish oxygen consumption equipment. Instead, reference was made to prior research^26^ to obtain preliminary results that helped provide qualitative descriptions of the findings. Although this method is persuasive, future studies should adopt more accurate measurement techniques. For instance, the use of electrom-yograms (EMGs) to record EMG values as descriptors of “energy costs”^45^ or measuring exercise consumption by monitoring the oxygen consumption of fish during exercise^64,65^, could provide valuable insights provided experimental conditions permit.

## Conclusion

In this study, the effects of flow stimulation training on the adjustment capacity of cultured grass carp to cope with running water were analyzed through comparative experiments. According to the results of entry frequency and residence time, both Tg and Cg fish showed a preference for low flow velocity ranges (2.5–3.5 BL/s), and the degree of preference of the Tg fish was higher than that of Cg. According to the movement path statistics of all experimental fish that completed the cycle, there were three types of routes. Route 1 mainly accounted for “wall-following” behavior and accounted for the consumption of minimal energy. Routes 2 and 3 represented an attempt to frequent high-velocity areas and led to the consumption of more energy. The Tg primarily selected route 1 and the Cg choose routes 2 and 3. This suggests that stimulating fish with an appropriate water flow velocity can enhance their sensitivity to current information and their ability to recognize low-velocity areas. This study provides valuable information and serves as a reference for enhancing fish survival rates through stock enhancement initiatives. The training practices suggested in this study, can be implemented before the stock enhancement initiatives and can potentially benefit fish to adapt to the complex aquatic environments of natural rivers earlier; thus, enhancing their ability to forage, avoid enemies, and migrate.

## Data Availability

The data that support the finding of this study are available on request from the corresponding author.
